# Quality assessment of clinical practice guidelines for chronic kidney disease: a systematic review

**DOI:** 10.1186/s12882-019-1387-x

**Published:** 2019-05-28

**Authors:** Jorge Coronado Daza, Robin W. M. Vernooij, Karla Salas, Dimelza Osorio, Gerard Urrútia Cuchí

**Affiliations:** 1 Facultad de Medicina, Universidad de Cartagena, Cartagena, 130014 Colombia; 2 Nefrologia y Diálisis SAS, Cartagena, 130001 Colombia; 3 Iberoamerican Cochrane Centre, 08041 Barcelona, Spain; 4 Biomedical Research Institute Sant Pau, 08025 Barcelona, Spain; 5 Hospital de la Santa Creu i Sant Pau, 08041 Barcelona, Spain; 6 University Hospital Vall d’Hebron, 08035 Barcelona, Spain; 7 CIBER Epidemiología y Salud Pública (CIBERESP), Barcelona, Spain

**Keywords:** Chronic kidney disease, Clinical practice guidelines, Quality assessment, Early diagnosis

## Abstract

**Background:**

Chronic kidney disease (CKD) is a worldwide public health problem. Clinical practice guidelines (CPGs) are being developed and implemented in order to improve clinical practice related to the detection and treatment of CKD. The objective of our study was to evaluate the quality of CPGs regarding the CKD and to examine whether there are factors which influence their quality.

**Methods:**

A systematic search was conducted to identify all CPGs regarding the early diagnosis and treatment of CKD. The CPGs quality were evaluated by three reviewers using the AGREE II instrument to decide if the guidelines are recommended for their use in clinical practice.

**Results:**

In total, 13 CPGs were identified: five from America, six from Europe, one from Asia, and one from Oceania. Five CPGs were recommended for their use in clinical practice; since all their domains achieved the medium or high category. Furthermore, six CPGs were recommended with modifications, as the stakeholders’ involvement, applicability, and editorial independence domains were evaluated as low category. These domains, as well as the rigor of the development domain, reached the very low category in those CPGs that were not recommended for its use in clinical practice. In all CPGs, the domains with the lowest average were the stakeholder involvement and the applicability. When comparing the domains of the CPGs according to the origin, type of developer group, the checklist used during the development and the publication period, a significantly higher average in the domain stakeholder involvement was found in the CPGs from Asia and Oceania compared to the ones in Latin America. Additionally, a significantly higher average in the applicability domain was found in the CPGs developed by CPGs developer organizations compared to those developed by medical societies.

**Conclusions:**

In total, 85% of the CPGs regarding CKD were recommended or recommended with modifications. The stakeholder involvement and applicability domains are assessed in the low category, which might affect the CPGs implementation. In order to save resources in low- and middle-income countries, an adaptation of the recommended CPGs should be considered.

**Electronic supplementary material:**

The online version of this article (10.1186/s12882-019-1387-x) contains supplementary material, which is available to authorized users.

## Background

Chronic kidney disease (CKD) is considered since 2005 a worldwide public health problem. Furthermore, since 2007, all countries have been urged to adopt actions for the control of CKD [1, 2]. The interest in establishing CKD as a public health issue was crucial to establish control measures and stopping the increase of CKD incidence. However, to date, there is no evidence stating that the use of control strategies has contributed to a decrease in the incidence of CKD. For instance, in the United States of America (USA), the incidence of CKD increased from 353 per million population (pmp) in 2005 to 370 pmp in 2014 [3].

One strategy to tackle the problem has been the development of CPGs for use of the stakeholders (physicians, patients, educators, health care providers, and government regulatory agencies). The main objectives of CPGs are the identification of the population at risk of CKD, early detection, and strategies to avoid the progression of the disease. To achieve these objectives, appropriate stakeholders must get involved in the development process of CPGs, as recommended by the AGREE (Appraisal of Guidelines for Research & Evaluation) and its updated version, AGREE II [4, 5]. The AGREE II instrument was also created to help achieving adequate reporting of the development process and presentation of CPGs.

In two recent systematic reviews the quality of CPGs addressing CKD has been analysed. The AGREE II domains with the lowest scores are stakeholder’s involvement and applicability [6, 7]. This is similar to CPGs evaluations in other diseases [8–10].

In this study, we present a quality assessment of CPGs regarding the early detection and management of CKD. Additionally, we evaluated whether there was a difference in the quality according to the region where the CPGs were developed, type of developer organization, self-assessment process in its development, and publication period.

## Methods

We included national and regional CPGs for the early detection and management of CKD. We included CPGs which included: 1) recommendations based on systematic evidence synthesis; 2) employing a grading system to rate the quality of evidence, 3) published in English, Spanish or languages that were feasible to translate for the authors; and 4) published between 2008 and 2016.

### CPGs identification

A systematic search was conducted in the main databases of organisations that develop or compile CPGs including: the Turning Research Into Practice (TRIP) database, National Guideline Clearinghouse (via https://www.ahrq.gov/topics/national-guideline-clearinghouse-ngc.html), National Institute for Health and Care Excellence (NICE), Scottish Intercollegiate Guidelines Network (SIGN), Guidelines International Network (G-I-N), and the National Health System library of Spain. An additional search was conducted in the international nephrology societies or associations webpages of each country or region. Additionally, a systematic search was performed in MEDLINE and EMBASE, investigating the medical terms headers related to CKD, applying CPG filters.

### Clinical practice guidelines assessment

Three authors independently (JC, RV, KS) assessed the quality of each CPG, under the guidance of an expert in Investigation Methodology (DO). The AGREE II instrument [5], which consists of 23 items organized in six domains, followed by 2 items of global score (overall assessment), was used. Each domain embraces a unique dimension in the CPG quality: scope and purpose, stakeholder(s) involvement, clarity of presentation, rigour of development, applicability, and editorial independence. The overall assessment includes a score for the general quality of the CPG and an assessment whether it is recommended for use in clinical practice. Each item was assessed using a 7-point scale (from 1 “strongly disagree” to 7 “strongly agree”), even if it was not applicable. The domain scores was expressed as a percentage over the highest possible score using the following formula:

$$ \frac{\mathrm{Obtained}\ \mathrm{score}-\mathrm{Minimum}\ \mathrm{possible}\ \mathrm{score}}{\mathrm{Maximum}\ \mathrm{possible}\ \mathrm{score}-\mathrm{Minimum}\ \mathrm{possible}\ \mathrm{score}} $$

The final score is the sum of the total scores assigned to each domain by each reviewer. The maximum possible score is 7 (strongly agree), multiplied by the number of items in the domain and the number of reviewers. The minimum possible score is 1 (strongly disagree) multiplied by the number of items in the domain and the number of reviewers. For the overall assessment, a score from 1 to 7 was consigned, as well as a recommendation regarding the use of the CPG in clinical practice classified as: recommended, recommended with modifications, and not recommended.

### Data analysis

Descriptive statistics were applied to analyse every domain (percentage; mean and standard deviation; median and interquartile range). The mean of the domains score was categorized as high (≥80%), medium (60–79%), low (40–59%), or very low (≤40%). The overall mean of each of the CPGs domains were compared using Student’s t-tests for independent samples (the test was two-tailed, and statistical significance was considered for *P*-values of less than 0.05), according to: CPG region, developer group, use of a self-assessment instrument, and publication period (2008–2011 versus 2012–2016).

The degree of agreement between reviewers was determined by the measurement of intraclass correlation coefficient (ICC) and its 95% confidence interval. An ICC of > 0.9 was considered “very good”, between 0.71 and 0.9 “good”, between 0.51 and 0.7 “moderate”, between 0.31 and 0.5 “fair”, and < 0.31 “poor” or “non-existent”.

## Results

**Fig. 1:**
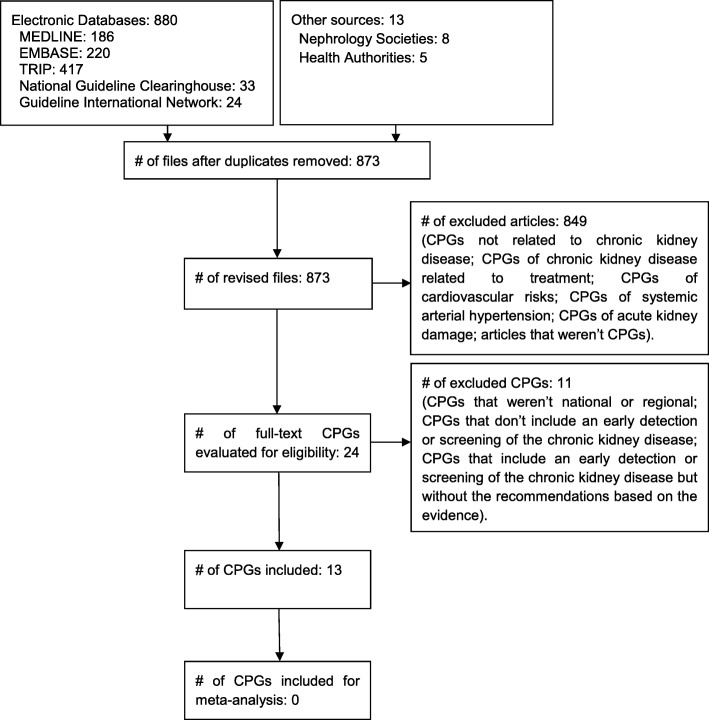
Flowchart of bibliographical research

The systematic search retrieved 893 records. After deleting duplicates and reviewing titles and abstracts, we identified 24 CPGs that were potentially eligible (Fig. 1). Consequently, after the full-text evaluation, 11 CPGs were excluded because they did not concern national CPGs, did not include recommendations regarding screening or early diagnosis of CKD, or did not present recommendations based on the evidence (Additional file 1). Finally, 11 national CPGs and two regional CPGs were included. The CPGs included were from Scotland [11], Spain [12], Netherlands [13], Mexico [14], Argentina [15], Chile [16], Malaysia [17], England [18], Italy [19], Sociedad Latinoamericana de Nefrología e Hipertensión (SLANH) [20], Australasia [21], USA [22] and United Kingdom (NICE guideline) [23].

General characteristics of the 13 included CPGs are presented in Table 1. Six CPGs were developed in Europe (46.2%), four in Latin America (30.7%), one in Asia (7.7%), one in Oceania (7.7%), and one in the USA (7.7%). Eight CPGs (61.5%) were published between 2008 and 2011 and five between 2012 and 2016 (38.5%). Regarding the language, five CPGs were written in Spanish (38.4%), six in English (46.2%), one in Italian (7.7%), and one in Dutch (7.7%). Eight CPGs (61.5%) were issued by organizations that develop CPGs, three (23.1%) by medical societies, and two (15.4%) by medical societies with an organization dedicated to developing CPGs (15.4%). Concerning the CPG developer panel, 12 (92%) included nephrologists, four (30.7%) experts in methodology, and four (30.7%) the target population. During the CPGs development process, only three CPGs (23.1%) used a checklist to verify if they were fulfilling the requirements that a CPG should have. The system used to classify the quality of evidence and the strength of the recommendations differed among the included CPGs; three (23.1%) used the GRADE (Grading of Recommendations Assessment, Development and Evaluation) system, three used the CPG developers’ own system (23.1%), three used mixed systems (23.1%), two (15.4%) the SIGN (Scottish Intercollegiate Guidelines Network) tool, one (7.7%) used CTFPHC (Canadian Task Force on Preventive Health Care) system, and one did not specify the used system (7.7%). Five CPGs (38.5%) were adapted from international CPGs: all four Latin America and one European.

**Table 1:** General characteristics of the included guidelines

Guidelines name	Country	Year of publication	Language of publication	Type of Guideline Developers	The GDG include nephrologists	The GDG include methodologists	The GDG include target population	Checklist	Grading system	Adapted
Diagnosis and Management of Chronic Kidney Disease. A National Clinical Guideline	Scotland	2008	English	^a^Organization	YES	NO	YES	NO	SIGN	NO
Guías SEN para el manejo de la Enfermedad Renal Crónica (ERC) Avanzada y Pre-Diálisis	España	2008	Spanish	^b^Medical society	YES	NO	NO	NO	Adapted of CTFPHC	NO
Richtlijn voor de behandeling van patiënten met Chronische Nierschade (CNS)	Netherlands	2009	Dutch	Medical society	YES	NO	NO	NO	Not specified	NO
Prevención Diagnóstico y Tratamiento de la ERC Temprana	México	2009	Spanish	Organization	YES	YES	NO	NO	Several	YES
Guía de Práctica Clínica sobre Prevención y Detección Precoz de la ERC en Adultos en el Primer Nivel de Atención	Argentina	2010	Spanish	Medical society and organization	YES	NO	NO	NO	SIGN	YES
Guía Clínica Prevención de ERC	Chile	2010	Spanish	Organization	YES	NO	NO	NO	Ministry of Health	YES
Management of chronic kidney disease in adults	Malaysia	2011	English	Organization	YES	YES	NO	NO	Modified SIGN; CTFPHC	NO
Renal Association Clinical Practice Guideline on Detection Monitoring and Management of Patients with CKD	England	2011	English	Organization	YES	NO	NO	AGREE	GRADE	NO
Identificazione, prevenzione e gestione della malattia renale cronica nell’adulto	Italy	2012	Italian	Organization	YES	YES	YES	Checklist: SNLG-ISS	Several	YES
Guías Latinoamericanas de Practica Clínica Sobre la Prevención, Diagnostico y Tratamiento de los Estadios 1–5 de la ERC	Latino América	2012	Spanish	Medical society and organization	YES	NO	NO	NO	GRADE	YES
KHA-CARI Guidelines: Chronic Kidney Disease	Australasia	2013	English	Organization	YES	NO	YES	NO	GRADE	NO
Screening, Monitoring, and Treatment of stage 1 to 3 Chronic Kidney Disease: A Clinical Practice Guideline From the American College of Physicians	United States	2013	English	Medical society	NO	YES	NO	NO	ACP	NO
Chronic kidney disease	United Kingdom	2014	English	Organization	YES	NO	YES	NICE	NICE	NO

^a^Government agencies, disease-specific organizations, non-profit health delivery systems, guidelines development agencies; ^b^Professional associations; *GDG* Guideline Development Group, *AGREE* Appraisal of Guidelines for Research & Evaluation, *SNLG-ISS* Sistema nazionale linee guida (National Guideline System), *SIGN* Scottish Intercollegiate Guidelines Network, *CTFPHC* Canadian Task Force on Preventive Health Care, *GRADE* Grading of Recommendations Assessment, Development and Evaluation, *KHA-CARI* Kidney Health Australia – Caring for Australasians with Renal Impairment, *ACP* American College of Physicians, *NICE* National Institute for Health and Clinical, *ERC* Enfermedad renal crónica (Chronic kidney disease)

### CPGs quality general assessment

**Fig. 2:**
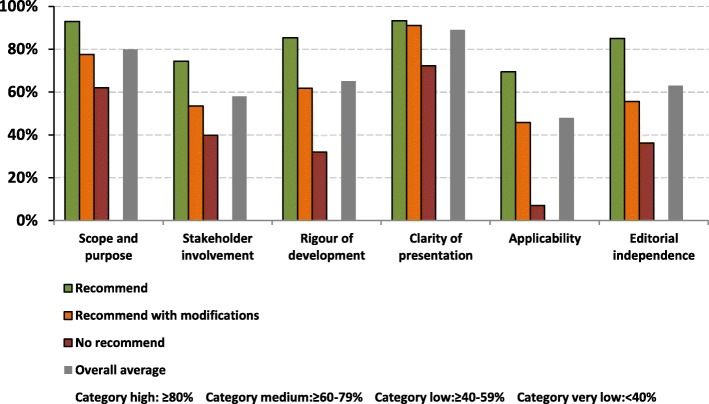
AGREE II Domain scores of Guidelines according to recommendation and category

The degree of agreement between the three reviewers was good, with an ICC of 0.88 (95% CI: 0.67–0.96) for the overall score. The CPG quality score varied from 3 to 7 and the reviewers recommended five CPGs (38.5%; CPG scores between 5 and 7: Scotland, Malaysia, Australasia, USA and United Kingdom). Six CPGs were recommended with modification (46.1%; CPG scores between 4 to 5: Mexico, Argentina, Chile, England, Italy and SLANH), and two CPGs were not recommended (15.4%; CPG scores between 3 to 4: Spain and Netherlands). The average score of each domain of all included CPGs and their respective recommendation can be observed in Table 2, and Fig. 2.

**Table 2:** Domain-standardized scores of each clinical practice guidelines, as assessed using the Appraisal of Guidelines Research Instrument

Guideline / Country or Region	Scope and Purpose (%)	Stakeholder Involvement (%)	Rigor of Development (%)	Clarity and Presentation (%)	Applicability (%)	Editorial Independence (%)	Overall Mean (%)	Recommendation
Diagnosis and Management of Chronic Kidney Disease. A National Clinical Guideline: Scotland	94.44	94.44	87.50	92.59	79.17	86.11	89.04	Recommended
Guías SEN para el manejo de la Enfermedad Renal Crónica Avanzada y Pre-Diálisis: España	64.81	27.78	22.92	81.48	11.11	19.44	37.92	Not recommended
Richtlijn voor de behandeling van patiënten met Chronische Nierschade (CNS): Netherlands	59.26	51.85	40.97	62.96	2.78	52.78	45.10	Not recommended
Prevención Diagnóstico y Tratamiento de la Enfermedad Renal Crónica Temprana: México	98.15	59.26	76.39	92.59	40.28	58.33	70.83	Recommended with modifications
Guía de Práctica Clínica sobre Prevención y Detección Precoz de la Enfermedad Renal Crónica en Adultos en el Primer Nivel de Atención: Argentina	90.74	59.26	73.61	88.89	52.78	66.67	71.99	Recommended with modifications
Guía Clínica Prevención de Enfermedad Renal Crónica: Chile	88.89	57.41	49.31	92.59	84.72	55.56	71.41	Recommended with modifications
Management of chronic kidney disease in adults: Malaysia	94.44	62.96	81.25	94.44	87.50	72.22	82.14	Recommended
Renal Association Clinical Practice Guideline on Detection, Monitoring and Management of Patients with CKD: England	59.26	38.89	50.69	90.74	47.22	61.11	57.99	Recommended with modifications
Identificazione, prevenzione e gestione della malattia renale cronica nell’adulto: Italy	50.00	48.15	63.19	88.89	30.56	55.56	59.06	Recommended with modifications
Guías Latinoamericanas de Practica Clínica Sobre la Prevención Diagnostico y Tratamiento de los Estados 1–5 de la Enfermedad Renal Crónica 2012: Sociedad Latinoamericana de Nefrología e Hipertensión Arterial (SLANH)	77.78	57.41	56.94	92.59	19.44	36.11	56.71	Recommended with modifications
KHA-CARI Guidelines: Chronic Kidney Disease: Australasia	92.59	74.07	81.94	90.74	61.11	77.68	79.69	Recommended
Screening, Monitoring, and Treatment of stage 1 to 3 Chronic Kidney Disease: A Clinical Practice Guideline From the American College of Physicians: United States of America (USA)	88.89	59.26	82.64	96.30	33.33	94.44	75.81	Recommended
Chronic kidney Disease: United Kingdom	94.44	81.48	93.06	92.59	86.11	94.44	90.35	Recommended
Mean (SD)	80.09 (17.12)	58.18 (17.38)	64.87 (21.19)	88.89 (8.97)	47.92 (30.39)	62.73 (22.3)		
Median (range)	88.89 (50–98.15)	59.26 (27.78–94.44)	73.61 (62.96–96.30)	92.59 (27.78–94.44)	45.83 (2.78–87.50)	61.11 (19.44–94.44)

### Domain 1: scope and purpose

This domain refers to the CPG general objective, the specific health aspects, and the population to whom this CPG is intended (items 1–3). The overall average score was 80.1% (median = 88.9%; interquartile range (IQR), 50 to 98.2%). Furthermore, 61.5% of the included CPGs had an average score of 80% or higher, which is considered high category. The average score of the recommended CPGs was 93, 77.5% for the CPGs recommended with modifications, and 62% for the CPGs that were not recommended.

### Domain 2: stakeholder involvement

This domain refers to the degree to which stakeholders have developed the CPG and the representation of the points of view of the intended users (items 4–6). The overall average was 58.2% (median = 59.3%; IQR, 27.8 to 94.4%). Furthermore, 15.3% of the CPGs were evaluated as high category, 15.3% as medium category, and 69.4% as low or very low category. The CPGs that were recommended had an average score of 74.4%, the CPGs recommended with modifications scored 53.4%, and the CPGs that were not recommended scored 39.8%.

### Domain 3: rigour of development

The rigour of development domain considers the process used to gather and synthesize the evidence, the methods to formulate the recommendations, and the updating process (items 7–14). The overall average score was 64.9% (median = 68.4%; IQR, 22.9 to 93.1%). Furthermore, 38.4% of the CPGs were considered within the high category, 23% within the medium category, and 38.6% within the low or very low category. The average score of the CPGs that were recommended for use in clinical practice was 85.3%, for CPGs recommended with modifications was 61.7%, and for those not recommended 31.9%.

### Domain 4: clarity of presentation

The clarity of presentation domain refers to the language, structure, and the CPGs format (items 15–17). The overall average was 88.9% (median = 92.59%; IQR, 62.96 to 96.3%). In total, 92.3% of the CPGs were considered within the high category. The average score of the CPGs recommended for use in clinical practice was 93.3%, for those recommended with modifications 91%, and for those not recommended 72.2%.

### Domain 5: applicability

This domain is related to the barriers and facilitators to its implementation, strategies to improve uptake, and resource implications when applying the CPGs (items 18–21). The overall average score was 47.9% (median = 43.75; IQR, 2.78 to 87.5%). In total, 23% of the CPGs were assessed as the high category, 15.3% as the medium category, and 61.7% as the low or very low category. The average score of the recommended CPGs was 69.4%, for those recommended with modifications 45.8%, and for those not recommended 6.9%.

### Domain 6: editorial independence

Finally, the editorial independence domain refers to the conflicts of interest of the panel members and the role of the funding body (items 22–23). The overall average was 62.7% (median = 59.72%; IQR, 19.44 to 94.44%). In total, 23% of the CPGs were in the high category, 30.7% in the medium category, and 46.3% in the low or very low category. The average score of the recommended CPGs was 85%, those recommended with modifications scored 55.6%, and those not recommended scored 36.1%.

### CPGs assessment according to recommendation

When comparing the AGREE II domains between the recommended CPGs and the CPGs recommended with modifications, we found a significant difference in the stakeholder involvement (74.4 ± 14.3 vs. 53.4 ± 8.2 respectively; *p* = 0.013), rigor of development (85.3 ± 5 vs. 61.7 ± 11.5 respectively; *p* = 0.002), and the editorial independence domains (85.0 ± 10 vs. 55.6 ± 10.4 respectively; *p* = 0.001). Between the recommended CPGs and the CPGs that were not recommended there was a statistically significant difference in all domains. A statistically significant difference was found between the CPGs recommended with modifications compared to those not recommended in the rigor of development (61.7 ± 11.5 vs. 31.9 ± 12.3 respectively; *p* = 0.02) and clarity of presentation domains (91.1 ± 1.8 vs. 72.2 ± 13.1 respectively; *p* = 0.006).

### CPGs assessment according to subgroups

The overall averages and the domain score of the included CGPs according to each subgroup are described as follows and resumed in Table 3.

**Table 3:** AGREE II domain scores of Guidelines according to different subgroups

Subgroup	Scope and Purpose Mean ± SD	Stakeholder Involvement Mean ± SD	Rigor of Development Mean ± SD	Clarity and Presentation Mean ± SD	Applicability Mean ± SD	Editorial Independence Mean ± SD	Overall Mean ± SD
Region							
Latinoamérica	88.89 ± 8.42	58.34 ± 1.07	64.06 ± 13.06	91.67 ± 1.85	49.31 ± 27.32	54.17 ± 12.93	67.74 ± 7.36
Europe	70.37 ± 19.24	57.10 ± 25.64	59.72 ± 27.14	84.88 ± 11.5	42.83 ± 34.57	61.57 ± 26.73	62.74 ± 22.13
Asia+Oceania	93.52 ± 1.31	68.52 ± 7.85	81.60 ± 0.49	92.59 ± 2.62	74.31 ± 18.66	74.95 ± 3.86	80.91 ± 1.73
Developers							
Medical societies	70.99 ± 15.75	46.30 ± 16.46	48.84 ± 30.63	80.25 ± 16.7	15.74 ± 15.8	55.56 ± 37.58	52.94 ± 20.12
Organization	84.03 ± 18.49	64.58 ± 18.05	72.92 ± 16.59	91.90 ± 1.7	64.58 ± 22.89	70.13 ± 14.9	74.69 ± 13.00
Medical societies and organization	84.26 ± 9.17	58.33 ± 1.31	65.28 ± 11.79	90.74 ± 2.62	36.11 ± 23.57	51.39 ± 21.61	64.35 ± 10.80
Self-assessment							
Yes	67.90 ± 23.45	56.17 ± 22.4	68.98 ± 21.77	90.74 ± 1.85	54.63 ± 28.51	70.37 ± 21.03	68.13 ± 19.27
No	85.00 ± 13.31	60.37 ± 16.71	65.35 ± 21.68	88.52 ± 9.83	47.22 ± 30.85	61.93 ± 22.67	68.07 ± 16.44
Year of publication							
2008–2011	81.25 ± 16.98	56.48 ± 19.47	60.33 ± 22.67	87.04 ± 10.52	50.70 ± 32.32	59.03 ± 19.23	65.80 ± 17.60
2012–2016	80.74 ± 18.36	64.07 ± 13.46	75.55 ± 14.97	92.22 ± 2.75	46.11 ± 27.12	71.65 ± 25.48	71.72 ± 14.98
Recommendation							
Recommended	92.96 ± 2.41	74.44 ± 14.25	85.28 ± 4.99	93.33 ± 2.11	69.44 ± 22.76	84.98 ± 9.96	83.41 ± 6.19
Recommended with modifications	77.47 ± 19.08	53.40 ± 8.23	61.69 ± 11.46	91.05 ± 1.82	45.83 ± 22.46	55.56 ± 10.39	64.17 ± 7.97
Not recommended	62,04 ± 3.93	39,81 ± 17.02	31,94 ± 12.77	72,22 ± 13.09	6,94 ± 5.89	36,11 ± 23.57	41.51 ± 5.07

### CPGs assessment according to the region

Out of the six CPGs developed in Europe, two were recommended (Scotland and United Kingdom), two recommended with modifications (England and Italy), and two were not recommended (Spain and Netherlands). The four Latin American CPGs (Mexico, Argentina, Chile and SLANH) were all recommended with modifications. The CPGs from Asia (Malaysia), Oceania (Australasia) and USA were all recommended. When comparing the domain scores of the CPGs from Asia and Oceania with the Latin American CPGs, we found a statistically significant difference in the stakeholder involvement domain (68.5 ± 7.9 vs. 58.3 ± 1 respectively; *p* = 0.04), however, when comparing with the European CPGs, no difference was found (68.5 ± 7.9 vs. 57.1 ± 25.6 respectively; *p* = 0.57).

In Latin American CPGs, the domains with the lowest scores were stakeholder involvement, applicability, and editorial independence. Similarly, in European CPGs, the domains with the lowest scores were stakeholder involvement and applicability. When comparing Latin American and European CPGs, we found no significant difference in the AGREE II domains.

### CPGs assessment according to the types of development groups

In total, three CPGs were developed by medical societies (Spain, Netherlands and USA), two were developed jointly by medical societies and organizations that develop CPGs (Argentina and SLANH) and the remaining eight CPGs by organizations responsible for developing CPGs.

For the organizations that develop CPGs and medical societies, the domains with the lowest scores were stakeholder involvement and applicability. When comparing these domains between the organizations that develop CPGs and medical societies, no statistically significant difference was found in the stakeholder involvement (64.6 ± 18.1 vs. 46.3 ± 16.5 respectively; *p* = 0.16) domain, however, we found a difference in the applicability domain (64.58 ± 22.9 vs. 15.74 ± 15.8 respectively; *p* = 0.009).

### CPGs assessment according to the self-assessment during the process of development

Three CPGs (23%) used a checklist during the development process to verify if they were fulfilling reporting requirements (England, Italy and United Kingdom). The overall score of the CPGs that self-assessed the fulfillment of the requirements was 68.1 ± 19.3 compared to the overall score of 68.1 ± 16.4 for the CPGs that did not self-asses their reporting (*p* = 0.10). In both groups, the domains with the lowest scores were stakeholder involvement and applicability with no significant statistical difference between them.

### CPGs assessment according to the time period of publication

For the period between 2008 and 2011, eight CPGs (61.5%) were published, out of which two were recommended, four were recommended with modifications, and two were not recommended. In the time frame between 2012 and 2016, five CPGs (38.5%) were published, out of which three were recommended and two were recommended with modifications.

The overall average score of the CPGs published in the period between 2008 and 2011 was 65.8 ± 17.6 and for the CPGs published in the period between 2012 and 2016 it was 71.7 ± 15 (*p* = 0.55). In both periods, the domains with the lowest scores were stakeholder involvement and applicability with no significant statistical difference between them.

### Synthesis of recommendations for chronic kidney disease

We identified general recommendations for CKD in the included CPGs: early diagnosis, investigations or diagnostic test, interventions for slowing the progression and criteria for referral to the nephrologist (Table 4). For the early diagnosis of CKD, all CPGs recommended to study CKD in patients with diabetes mellitus and systemic arterial hypertension. Furthermore, the majority of the CPGs stated that patients with cardiovascular diseases, systemic diseases that affect the kidney and family history of end-stage kidney disease must be investigated.

**Table 4:** General guidelines recommendations for chronic kidney disease

	Number	Percent
Screening for Chronic Kidney Disease (CKD)		
Diabetes mellitus	13	100
Hypertension	13	100
Cardiovascular disease	11	85
Family history of end-stage kidney disease	10	77
Multisystem diseases with potential kidney involvement	8	62
Structural renal tract disease	8	62
Chronic use of nephrotoxic drugs	6	46
Proteinuria	6	46
Aged over 60 years	4	31
Isolated Hematuria	3	23
Low socioeconomic status	3	23
Cigarrete smoking	3	23
Obesity	1	8
Investigations for CKD		
Creatinine-based equations for estimation renal function	13	100
^a^MDRD	11	85
Cockcroft Gault	6	46
^b^CKD-EPI	4	31
Albuminuria	13	100
Proteinuria	10	77
Hematuria	5	39
Renal ultrasound	2	15
Interventions for slowing the rate of progression of CKD		
Optimal blood pressure range	13	100
Use of angiotensin converting enzyme inhibitors or angiotensin receptor blockers	13	100
Optimal proteinuria reduction	12	92
Optimal weight	12	92
Smoking cessation	12	92
Lipid lowering with statin therapy	12	92
Sodium restriction	10	77
Exercise	10	77
Optimal glycemic control	9	69
Patient education	5	39
Moderate intake of alcohol	4	31
Referral to the nephrologist		
Statement of criteria for referral	11	85
Reduced glomerular filtration rate or creatinine clearance	11	100
< 30 mL/min/1.73m^2^	8	73
< 60 mL/min/1.73m^2^	2	18
< 45 mL/min/1.73m^2^	1	9
Proteinuria or albuminuria	11	100
Glomerular hematuria	8	73

^a^Modification of Diet in Renal Disease

^b^Chronic Kidney Disease Epidemiology Collaboration

To evaluate the presence of CKD, all CPGs recommended estimating the glomerular filtration with the formulas based in the serum creatinine and to measure the albuminuria. The formula most used to estimate the glomerular filtration is MDRD (Modification of Diet in Renal Disease).

To avoid the progression of CKD, all CPGs recommended stabilizing the arterial pressure and using angiotensin converting enzyme inhibitors or angiotensin II receptors blockers. The majority of the CPGs recommended controlling proteinuria, stabilize weight, avoid smoking, and controlling lipids.

Eleven (85%) included CPGs reported criteria to obtain a referral to the nephrology department, this concerns especially patients with a low estimated glomerular filtration and proteinuria or albuminuria. The majority of the CPGs (73%) recommend a referral to a nephrologist when the estimated glomerular filtration is below 30 mL/min/1.73m^2^.

## Discussion

The 13 CPGs focused on the early detection of the CKD and included in this study represent the current scenario worldwide, since we included CPGs published between 2008 and 2016 in different languages, with a geographical variation over all continents.

Our review shows that the majority of CPGs focused on the early diagnosis of the CKD are of good quality and developed by organizations that develop CPGs. The recommended CPGs have a medium to high score in all the domains. The CPGs recommended with modifications only have a medium to high score on scope and purpose, rigor of development and clarity of presentation domains. However, the domains on the stakeholder involvement, applicability and editorial independence did not reach a score of medium category.

### Comparison with the existing literature

In general, all CPGs have their lowest averages in the stakeholder involvement, applicability and editorial independence domains, which could partly explain why the incidence of the CKD hasn’t decreased [3]. In the review by López-Vargas et al., regarding the CPGs published between 2002 and 2011, similar results have been found [6]. The domains with the lowest average were stakeholder involvement, applicability, and editorial independence. The review of Sekercioglu et al., regarding CPGs published between 2003 and 2015 that focused in the alterations in the bone mineral metabolism in CKD, reported as well that the domains with the lowest averages (all being in the category very low) were stakeholder involvement, applicability, and editorial independence [7]. Our results are similar to those found by Gagliardi and Brouwers; they analyzed systematic reviews regarding different pathologies, which included CPGs published since 2008, finding that the three domains with the lowest scores were stakeholder involvement, applicability, and editorial independence. Among the factors associated with the applicability, there was a significantly higher average in the CPGs elaborated by groups that develop CPGs. Unlike our study, they found a significantly higher average in the CPGs that were published between 2010 and 2012. In their conclusions, they state that the applicability of the CPGs has not increased in relation to the those published before 2008 and that the cost to elaborate CPGs is not being rewarded by their applicability [24].

### Strategies for implementation the CPGs

The findings of our review and those of others point to the same weakness in the current CPGs. Due to the weaknesses in the implementation, the recommendations with the best evidence are not being used fully in the daily practice. This, as we mentioned before, can be one of the determining factors in the increasing incidence of CKD, despite the existing recommendations in the CPGs regarding prevention. To enhance the applicability of the CPGs, it has been recommended, among other strategies, to endow clinics with instruments that facilitate the implementation of these CPGs. The idea is to simplify the recommendations to be executed and to achieve the facilitation of the application and understanding of the patients and the healthcare providers [25, 26]. In a recent study, all ideal characteristics for a CPGs’ implementation instrument were explored, and identified 12 items. Among these items, the following were highlighted: identification of target users; involvement of target users were in the tool development; and conduction of pilot-test of the tool in target users [27]. Kastner et al. found that the factors associated with the implementation of the CPGs are the ones related to the creation of the content of the CPG and the proper communication of its content [28]. In relation to the content, they consider the stakeholder involvement and the feasibility important among others, similarly to our study. Another study was executed to develop a model based in the evidence for the implementation of the CPGs of clinical practice. Based on their results, the GUIDE-M (Guideline Implementability Decision Excellence Model) was created with the purpose of helping organizations that developed CPGs to create CPGs with recommendations easier to implement, facilitate the users’ adoption and to motivate researchers for deeper investigations in the topic [29].

On the other hand, the AGREE II instrument has been used more as an instrument for assessing CPGs’ quality despite the fact that it was also created to help achieve the requirements of the development and presentation of the CPGs. In our study, we reported that only 23.1% (3/13) of the CPGs, performed a process of self-assessment or compliance verification of the requisites needed for the development of the CPG, hence this could have influence the low scores of the stakeholder involvement and applicability domains. In 2016 the AGREE Reporting Checklist was published, which seeks, among its objectives, to help the CPGs’ developers take into account each of the quality requirements proposed in AGREE II [30]; with the use of this instrument, it would be possible in future CPGs to overcome the weaknesses found in our study and in the other ones aforementioned. Within the AGREE platform, a research project is registered to create a new instrument that complements AGREE II, which has been named AGREE-REX (**A**ppraisal of CP**G**s **RE**search and **E**valuation – **R**ecommendations **EX**cellence) [31]. The purpose of this project is to create an instrument that is useful for the development, report and evaluation activities related to the credibility, optimization, reliability and implementability of the CPGs recommendations. It is expected to be ready for publication in the following years.

### Implications for clinical practice

The general recommendation especially in low- and middle-incomes countries, is the adaptation of high quality CPGs to their context by using recommendations based in the best evidence available and focused in the early diagnosis of the CKD [32]. Ours results allow the endorsement of the CPGs developed in Scotland, Malaysia, Australasia and the United Kingdom (NICE guideline) to support other developer groups to create their own CPGs or adapt them to their context. For this last purpose, the CPG from the USA is not endorsed due to a low and very low score on the stakeholder involvement and applicability domains, respectively.

We considered that the adapted CPGs from Mexico, Argentina, Chile, Italy and SLANH have recommendations of utility in their context but they require an improvement in the domains stakeholder involvement and applicability in future updates so they can truly contribute to make an impact over the incidence of the CKD.

### Strengths and weaknesses

As far as we know, our study represents the first analysis published on CPGs quality over the last 8 years worldwide, with no restriction regarding language, focused on the early diagnosis of the CKD including recommendations based on the latest evidence. Among the strengths, we included a systematic search of the published CPGs, a high degree of agreement among reviewers, a great expertise of methodological experts in the evaluation of CPGs using the AGREE II instrument. Additionally, two of the reviewers are specialists in the area of nephrology. We highlight as a strength the fact that the nephrologist reviewers work in different continents and are native from different countries.

Among the weaknesses, we mentioned that our inclusion criteria only admitted potentially high quality CPGs, which can be a selection bias. Although no quality threshold has been established in the AGREE II instrument, we accepted as satisfactory an average score of 60% or more in the domains, which may be argued by other authors.

## Conclusions

The majority of the CPGs focused on the early diagnosis of the CKD are recommended for their use in clinical practice. However, in clinical practice we notice an increase in incidence of CKD, which suggests that the recommendations probably are not being properly applied. We found that the AGREE II domains with the lowest average in all CPGs are the stakeholder involvement and the applicability, which may be factors influencing implementation. The cost to elaborate CPGs is not being rewarded by their implementation, for this reason, the general recommendation especially in low- and middle-incomes countries, is the adaptation of high quality CPGs to their context.

## Additional file

Additional file 1: Summary of the excluded guidelines and exclusion criteria. We reported the titles of the guidelines that were excluded and the inclusion criteria they failed to meet. (DOCX 19 kb)

## Abbreviations

AGREE: Appraisal of Guidelines for Research & Evaluation
CKD: Chronic kidney disease
CPG: Clinical practice guideline
NICE: National Institute for Health and Care Excellence

## Authors’ information

JC is a physician specialized in Internal Medicine and Nephrology, with a Master degree in Clinical Epidemiology. He is a teacher in the Faculty of Medicine of the Universidad de Cartagena, Colombia and leader of the Alta Tension research group; also, is a PhD candidate in Methodology of Biomedical Research, Universidad Autónoma de Barcelona, Spain.
