# Supplementary material for: Quality assessment of clinical practice guidelines for chronic kidney disease: a systematic review
Source: BMC Nephrol. 2019 May 28;20:192. doi: 10.1186/s12882-019-1387-x (PMC6540562; doi:10.1186/s12882-019-1387-x)
Supplement: Supplementary file 1 — Summary of the excluded guidelines and exclusion criteria. We reported the titles of the guidelines that were excluded and the inclusion criteria they failed to meet. (DOCX 19 kb) [file 12882_2019_1387_MOESM1_ESM.docx]

| **Additional file 1 Summary of the excluded guidelines and exclusion criteria** | | | | | |
| --- | --- | --- | --- | --- | --- |
| **Guidelines name** | **Origin** | **Year** | **Country or region** | **Grading system** | **Early detection of CKD** |
| Guidelines for the management of chronic kidney disease | Canada | 2008 |  |  | **⊗** |
| Evidence-based Practice Guideline for the Treatment of CKD | Japan | 2009 |  |  | **⊗** |
| Guía clínica para identificación, evaluación y manejo inicial del paciente con enfermedad renal crónica en el primer nivel de atención | Perú | 2010 |  | **⊗** |  |
| Maladie Rénale Chronique de l’adulte | France | 2012 |  | **⊗** |  |
| KDIGO 2012 Clinical Practice Guideline for the Evaluation and Management of Chronic Kidney Disease | United States of America | 2012 |  |  | **⊗** |
| Indian chronic kidney disease guidelines | Indian | 2013 |  |  | **⊗** |
| Management Chronic Kidney Disease | Michigan | 2014 | **⊗** |  |  |
| Chronic Kidney Disease - Identification, Evaluation and Management of Adult Patients | British Columbia | 2014 | **⊗** | **⊗** |  |
| *****VA/DoD Clinical practice guideline for the management of chronic kidney disease in primary care | United States of America | 2014 | **⊗** |  |  |
| Northern Ireland Guidelines for the Management of Chronic Kidney Disease (CKD) | Northern Ireland | 2015 |  | **⊗** |  |
| Guía de Práctica Clínica para el diagnóstico y tratamiento de la Enfermedad Renal Crónica | Colombia | 2016 |  |  | **⊗** |

*****Only include the population of Veterans Health Administration and Military Health System; CKD, Chronic Kidney Disease.

**⊗**Exclusion criteria
